# Spatially resolved measurement of helium atom emission line spectrum in scrape-off layer of Heliotron J by near-infrared Stokes spectropolarimetry

**DOI:** 10.1038/s41598-022-19747-8

**Published:** 2022-09-23

**Authors:** Tomoki Chatani, Taiichi Shikama, Yohei Ueno, Shinichiro Kado, Hayato Kawazome, Takashi Minami, Ryota Matoike, Minato Murakumo, Shinji Kobayashi, Shinsuke Ohshima, Akihiro Iwata, Tetsutaro Oishi, Akihiro Ishizawa, Yuji Nakamura, Hiroyuki Okada, Shigeru Konoshima, Tohru Mizuuchi, Kazunobu Nagasaki, Masahiro Hasuo

**Affiliations:** 1 Department of Mechanical Engineering and Science, Graduate School of Engineering, Kyoto University, Kyoto, 615-8540 Japan; 2 Institute of Advanced Energy, Kyoto University, Kyoto, 610-0011 Japan; 3 National Institute of Technology, Kagawa College, Kagawa, 769-1192 Japan; 4 Graduate School of Energy Science, Kyoto University, Kyoto, 610-0011 Japan; 5 National Institute for Fusion Science, Gifu, 509-5292 Japan

**Keywords:** Magnetically confined plasmas, Near-infrared spectroscopy

## Abstract

For plasma spectroscopy, Stokes spectropolarimetry is used as a method to spatially invert the viewing-chord-integrated spectrum on the basis of the correspondence between the given magnetic field profile along the viewing chord and the Zeeman effect appearing on the spectrum. Its application to fusion-related toroidal plasmas is, however, limited owing to the low spatial resolution as a result of the difficulty in distinguishing between the Zeeman and Doppler effects. To resolve this issue, we increased the relative magnitude of the Zeeman effect by observing a near-infrared emission line on the basis of the greater wavelength dependence of the Zeeman effect than of the Doppler effect. By utilizing the increased Zeeman effect, we are able to invert the measured spectrum with a high spatial resolution by Monte Carlo particle transport simulation and by reproducing the measured spectra with the semiempirical adjustment of the recycling condition at the first walls. The inversion result revealed that when the momentum exchange collisions of atoms are negligible, the velocity distribution of core-fueling atoms is mainly determined by the initial distribution at the time of recycling. The inversion result was compared with that obtained using a two-point emission model used in previous studies. The latter approximately reflects the parameters of atoms near the emissivity peak.

## Introduction

In fusion-related toroidal plasmas, emission spectroscopy is used for plasma control and machine protection by measuring impurity generation and transport, confinement mode transition, hydrogen isotope ratio, neutral density, and so forth^1,2^. These diagnostics have, however, a drawback that they can measure only the viewing-chord-integrated spectrum. To obtain a spatially resolved spectrum, additional methods such as computer-aided tomography (CT) with more than two directional observations using multiple viewing chords or active emission spectroscopy for detecting a neutral beam or laser-induced emission from the intersection between the beam and the viewing chord are required. The application of these methods will, however, become difficult in future fusion reactors owing to the limitation in the available port area^2,3^. It is thus desirable to develop an alternative method that is implementable by using a single diagnostic port.

A method that can satisfy this requirement is Stokes spectropolarimetry, which is widely used in the fields of astrophysics and ellipsometry^4–8^. It determines the polarization state of an emission line spectrum by measuring the Stokes parameters *I*, *Q*, *U*, and *V* of this spectrum. For the spectroscopy of toroidal plasmas, polarization is mainly induced by the spatial anisotropy produced by the existence of an external magnetic field. The field effect on the spectrum emerges as the Zeeman effect that induces the wavelength splitting of transitions among magnetic sublevels and wavelength-dependent polarization of the spectrum^9,10^. Stokes spectropolarimetry can then be used to measure the magnetic field from a spectrum^11–15^. For toroidal plasmas, it is possible to use Stokes spectropolarimetry as an inversion method for a chord-integrated spectrum on the basis of the correspondence between the given magnetic field profiles measured by other methods and the magnetic field effect emerging on the spectrum^16,17^. Compared to inversions for astrophysical plasmas^6,18,19^, instead of a given magenetic field profiles, the observation direction and available number of viewing chords are limited, and established models for the emissivity and velocity distribution profiles of the light emitting particles do not exist.

**Figure 1:**
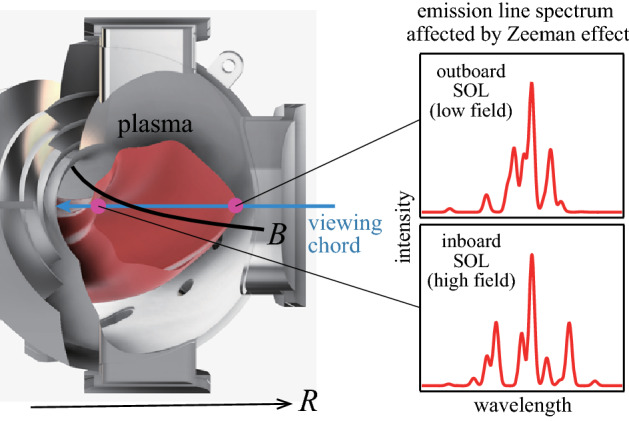
Schematic of typical observation geometry for diagnostics (ii). The illustrated spectra are those of HeI 2^3^S-2^3^P at 1 and 2 T observed in the direction perpendicular to the magnetic field without resolving polarization.

The inversion by Stokes spectropolarimetry has been mainly applied to atomic emission lines in boundary plasmas for three types of diagnostics: (i) to identify the location of a nearly localized emission existing on the viewing chord (one-point emission model)^16,20^, (ii) to decompose the chord-integrated spectrum into two spectra originating from the inboard and outboard scrape-off layers (SOLs) (two-point emission model)^17,21–29^, and (iii) to remove the effect of strong divertor emission reflected from the first walls (FWs) (synthetic emission model)^30,31^. Figure 1 schematically illustrates the typical observation geometry of diagnostics (ii). A chord-integrated spectrum is measured with a radial viewing chord and the spectra originating from the inboard and outboard SOLs are separated using the difference between their Zeeman effects produced by the radial magnetic field gradient. The viewing chord is nearly perpendicular to the magnetic field, and the field can be virtually determined by measuring only the linear polarization (Stokes parameters *I*, *Q*, and *U*).

The spatial resolutions of the above-mentioned diagnostics were, however, limited, because of the convoluted Doppler and Stark effects on the spectrum. These effects have comparable magnitudes to the Zeeman effect for the visible emission lines, and due to the lack of a model for their spatial profiles, it is difficult to distinguish these effects from the Zeeman effect. The degradation of the spatial resolution by these effects is an obstacle to the versatile application of the inversion. In this study, we demonstrated two measures to improve the spatial resolution: the observation of a near-infrared (NIR) emission line and the adoption of a simulation-based model for the emissivity and velocity distribution profiles. The former increases the relative magnitude of the Zeeman effect owing to the difference in the wavelength dependence of the Zeeman effect from that of the Doppler effect. On the other hand, the latter combined with the result of the former enables distinguishing the Zeeman, Doppler, and Stark effcts. As a result, a higher spatial resolution than those obtained in previous studies can be ontained for the measurement of the atomic emission lines and the results contribute to improved diagnostics of the above-mentioned quantities.

## Methods

### Wavelength dependences of Zeeman, Stark, and Doppler effects

The application of Stokes spectropolarimetry requires an observable Zeeman effect. The minimum field strength satisfying this requirement is approximately obtained as the value giving a wavelength shift by the Zeeman effect equivalent to the line width. In toroidal plasmas, the line width is dominated by the Stark and Doppler effects. Since the wavelength dependences of the Zeeman and Stark effects are different from that of the Doppler effect, the relative magnitude of the former two effects can be increased by observing an emission line of a longer wavelength^11,32,33^.

The Zeeman and Stark effects produce shifts in energy level, and when the energy level shift is small, the resultant shift in emission line wavelength can be approximated as

$$\delta \lambda = - \frac{{\lambda^{2} }}{hc}\delta E$$

where *δλ* and *δE* are the shifts in wavelength and transition energy, respectively, and *c* is the velocity of light. For a given *δE*, *δλ* is approximately proportional to λ^2^. The variation in *δE* with energy level is within a factor for the Zeeman effect for major hydrogen and helium atomic emission lines. On the other hand, for the Stark effect, it largely depends on the magnitude of the electric dipole moment pertaining to energy level. The Stark effect therefore can be mitigated by observing an emission line whose upper and lower energy levels have small electric dipole moments.

For the Doppler effect, the Doppler shift is given as

$$\delta \lambda_{{\text{D}}} = \frac{{v_{{{\text{vc}}}} }}{c}\lambda$$

where *v*_vc_ is the velocity of emitters in the viewing chord direction. The Doppler width produced by emitters having a Maxwellian *v*_vc_ distribution is given as

$$w_{{\text{D}}} = \frac{\lambda }{c}\sqrt {\frac{{(8\ln 2)k_{{\text{B}}} T}}{m}}$$

where *k*_B_ is the Boltzmann constant and *T* and *m* are the temperature and mass of emitters, respectively. For a given *T*, *δλ*_D_ and *w*_D_ are proportional to λ.

From a practical viewpoint, in addition to the above-mentioned factors, the observed spectrum is convoluted with the instrumental function of the spectrometer. Its wavelength dependence varies with the type of spectrometer used. For instance, for Czerny-Turner spectrometers, it is determined by the entrance slit width, diffraction limit, and aberration. The wavelength dependences of these factors are generally less than proportional to λ. As a whole, by observing an emission line of a longer wavelength and a small Stark effect, the relative magnitude of the Zeeman effect can be increased and the minimum field strength satisfying the requirement can be reduced. We measured a bright NIR helium atomic emission line HeI 2^3^S-2^3^P at 1083 nm whose Stark effect is negligible^34^. Its wavelength is not much longer than those of the visible lines, but it has advantages of transmittance to fused silica and being less susceptible to the effect of blackbody radiation from plasma-facing components and spectroscopic instruments. The line has also been widely employed for Stokes spectropolarimetry of astrophysical plasmas^35–37^.

### NIR spectropolarimetry experiments

**Figure 2:**
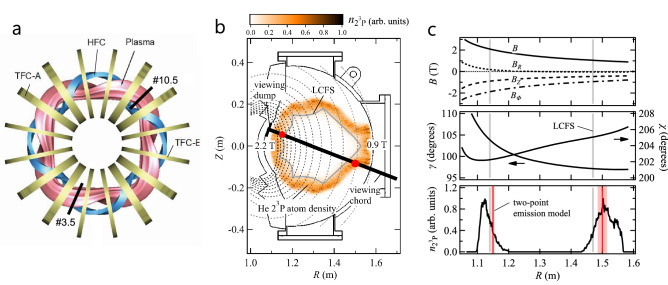
(**a**) Top view of Heliotron J with a helical field coil (HFC) and toroidal field coils (TFC-A and TFC-E). (**b**) #10.5 poloidal plane and viewing chord. The magnetic field strength of 0.9–2.2 T is shown with dashed lines. The relative 2^3^P excited helium atom density $n_{{2^{3} P}}$ obtained by simulation is shown with the color map and the emission locations and relative intensities determined using the two-point emission model are plotted with red circles. (**c**) Spatial profiles of magnetic field, *γ*, *χ*, and $n_{{2^{3} P}}$ (bin size is different from that of **b**) along the viewing chord. The grey lines show the locations of the LCFS and the red lines show the emission locations determined using the two-point emission model.

We conducted experiments in a medium size heliotron device Heliotron J^38^. The top view of the device is illustrated in Fig. 2a. A magnetic field of 1–2 T was produced with a helical field coil (HFC), two types of toroidal field coils (TFC-A and TFC-E), and vertical field coils (not shown in the figure). Spectropolarimetry was conducted along the #10.5 poloidal plane, where the field strength reaches maximum on the inboard side of the torus and monotonically decreases toward the outboard side (Fig. 2b). Helium-puffed deuterium discharges were produced with 240 kW ECH for approximately 200 ms. The toroidally averaged major and minor plasma radii were 1.2 m and 0.17 m, respectively, and the averaged field strength was 1.35 T at the magnetic axis. The plasma parameters measured by Thomson scattering in the #3.5 poloidal plane were *T*_e_ = 1 keV and *n*_e_ = 1 × 10^19^ m^−3^ at the core and *T*_e_ = 100 eV and *n*_e_ = 5 × 10^18^ m^−3^ at the last closed flux surface (LCFS).

For spectropolarimetry, a radial viewing chord tilted by 21° upward from the horizontal plane was used. To remove the effect of light reflected from FWs, a chevron-type viewing dump^39^ was installed at the opposite surface. The variation in magnetic field along the viewing chord is shown in Fig. 2c, where *γ* and *χ* are the polar and azimuthal angles, respectively. The variations in *γ* and *χ* are small and are in the ranges of *γ* = 97–105° and *χ* = 202–206°. Thus, the linear polarization spectra in the directions *χ* = 24.5° and 114.5° were simultaneously observed using a polarization beam splitter, and they mainly consist of the π and σ components, respectively, both at the inboard and outboard SOLs; we denote these spectra as *I*_0_ and *I*_90_, respectively.

An NIR Stokes spectropolarimetry system consisting of a polarization beam splitter (extinction ratio 3 × 10^–3^) with two collimators, bundled optical fibers, a Czerny-Turner type spectrometer (1 m focal length and 720 grooves/mm grating at second-order diffraction), and an InGaAs linear array was used to simultaneously measure *I*_0_ and *I*_90_ with a single viewing chord with a diameter of approximately 23 mm. In the NIR wavelength range, the photodetector dark noise increases compared with that in the visible wavelength range; thus, we adopted the following countermeasures to improve the signal-to-noise ratio of the spectra. (i) The throughput of the spectrometer was increased by reducing the light loss at the entrance slit of the spectrometer with the bifurcation of the light collected with the single viewing chord into seven optical fibers and aligning the fibers along the entrance slit both for *I*_0_ and *I*_90_. (ii) The statistical distributions of the dark noise were measured for each photodetector pixel and flawed pixels were excluded from measurements use. (iii) The spectra were acquired with an exposure time of 120 ms during the flattop phase of the discharge and averaged over ten discharges produced under identical operating conditions. The intensity and wavelength of the measured *I*_0_ and *I*_90_ were absolutely calibrated, and a NeI emission line spectrum of a glow discharge plasma was regarded as the instrumental function. The instrumental width was approximately 45 pm for both *I*_0_ and *I*_90_.

### Synthesis of chord-integrated emission line spectrum

We synthesized the chord-integrated *I*_0_ and *I*_90_ using a simulation result so that they reproduce the measured ones and regarded the simulation result as the inversion result. To reduce the computation time of the simulation, we assumed that the effect of helium gas puffing on the main deuterium plasma is small and carried out two independent simulations: (i) the EMC3-EIRENE calculation of the three-dimensional profiles of *T*_e_ and *n*_e_ for a pure deuterium plasma (background plasma), and (ii) the Monte Carlo particle transport calculation of two-dimensional profiles of the density $n_{{2^{3} P}}$ and velocity distribution *f*(*v*_vc_) of 2^3^P excited helium atoms in the #10.5 poloidal plane. In simulation (ii), *T*_e_ and *n*_e_ obtained by simulation (i) were used as fixed background plasma parameters and the FW recycling condition was semiempirically determined as the parameters of least-squares fitting. In the synthesis of the chord-integrated spectra, the Stokes parameters were calculated using a vector radiative transfer equation^40^ expressed as

1 $$\frac{dI(s,\lambda )}{{ds}} = \epsilon (s,\lambda ) - K(s,\lambda )I(s,\lambda )$$

where ***I*** = (*I*, *Q*, *U*, *V*)^T^ is the Stokes vector, *s* is a coordinate along the viewing chord, ***ε*** = (*ε*_*I*_, *ε*_*Q*_, *ε*_*U*_, *ε*_*V*_)^T^ is the emissivity vector, and *K* is the absorption matrix. For the present experimental condition, absorption is negligible owing to the relatively small plasma size and partial pressure of helium, and we set $K = 0$ as an approximation. Note that absorption is not negligible in a larger device operated at a higher partial pressure of helium^41^.

In simulation (i), an EMC3-EIRENE code customized for Heliotron J^42,43^ was used. The input parameters were set to the previously determined values except for the electron and ion thermal diffusion coefficient and edge electron density at *a/r* = 0.8, where *r* is the minor radius and *a* is that at the LCFS. The values of the input parameters are summarized in Table S1. The optimized two parameters are sensitive to the discharge condition and were determined so as to minimize the differences in *T*_e_ and *n*_e_ between the simulation results and the results of measurement by Thomson scattering in the #3.5 poloidal plane. These parameters were set to 2.5 m^2^ s^−1^ and 1.0 × 10^19^ m^−3^, respectively.

In simulation (ii), a general Monte Carlo algorithm was used in a 2d3v space. For a real space, the same *RZ* area in the poloidal plane of spectropolarimetry as in simulation (i) was used and the periodic boundary condition was assumed in the perpendicular direction. All the helium atoms were assumed to be produced by desorption from the FWs as the 2^1^S ground-state atoms having a “*v*^3^” *T*-Maxwell distribution^44^, which is satisfied when the influx and outflux at the surface are balanced. The velocity distribution is a function of the temperature *T* and the angular distribution was assumed to be the cosine distribution^45^. For the collisional processes in the plasma, only inelastic collisions with electrons are considered. The atoms are thus affected only by excitation and ionization and their velocities are kept constant. This is a crude approximation and the effects of other collisions such as the elastic momentum exchange collision with electrons will be addressed in a future study. From the given magnetic field profile and *f*(*v*_vc_) obtained by simulation (ii), *I*_0_ and *I*_90_ were calculated using Eq. (1). The Zeeman effect was evaluated using the first-order perturbation method^46^.

## Results and discussion

**Figure 3:**
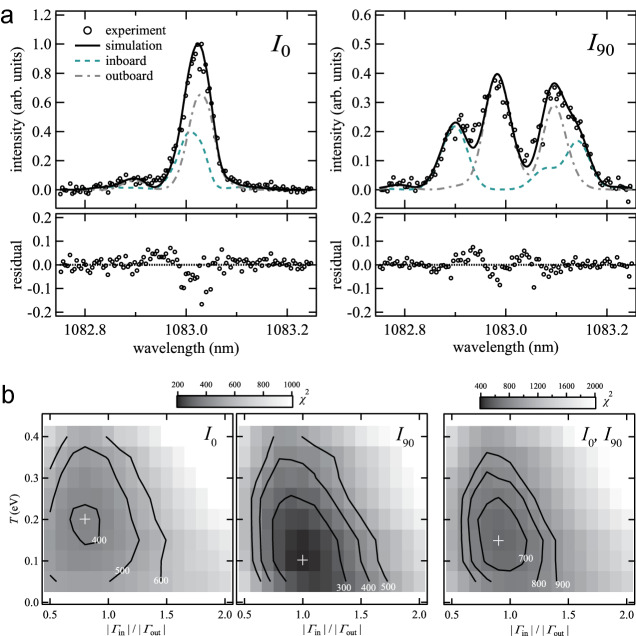
(**a**) *I*_0_ and *I*_90_ of a chord-integrated HeI 2^3^S-2^3^P emission line spectrum averaged over 10 discharges (#80303–312). The lines show the fitted spectra obtained by simulation. (**b**) *χ*^2^ distributions as functions of the parameters *T* and $|\Gamma_{{\text{in}}} /\Gamma_{{\text{out}}} |$ for fitting to *I*_0_, *I*_90_, and both *I*_0_ and *I*_90_. *T* and $|\Gamma_{{\text{in}}} /\Gamma_{{\text{out}}} |$ were varied at discrete step sizes of 0.05 eV and 0.1, respectively, and the cross marks show *χ*^2^ minima.

The measured *I*_0_ and *I*_90_ are shown in Fig. 3a. They consist of emission from inboard (~ 2 T) and outboard (~ 1 T) SOLs. Although the field strength of Heliotron J was smaller than those in the devices used in previous studies (2–8 T), the Zeeman effect on the *I*_90_ spectrum is conspicuous owing to the advantage of NIR spectropolarimetry. Note that in future fusion reactors, the field strength and its spatial gradient will be several times larger than those in Heliotron J. In this case, the relative magnitude of the Zeeman to Doppler effects will be further increased.

**Figure 4:**
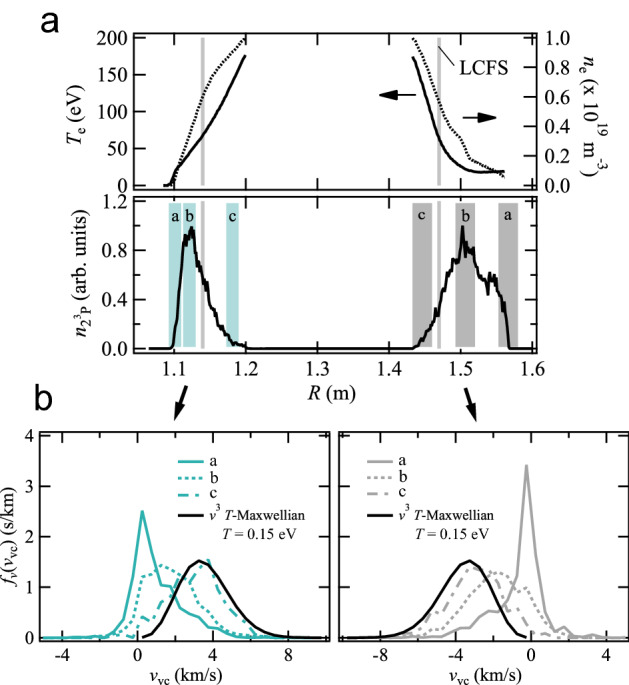
(**a**) $n_{{2^{3} P}}$ along the viewing chord. (**b**) Spatially resolved *f*(*v*_vc_) at shaded regions a, b, and c. The solid black lines indicate the “*v*^3^” *T*-Maxwellian distribution at *T* = 0.15 eV, which is the assumed initial distribution at the FWs.

We determined the recycling condition, which is an uncertain factor in simulation (ii), by setting *T* and the recycling flux as the parameters and performing a least-squares fitting of the synthesized *I*_0_ and *I*_90_ to the measured ones. Owing to the limitation in the number of viewing chords, we made an approximation that the recycling flux is uniform over the inboard ($R \le 1.3$ m) and outboard ($R > 1.3$ m) FWs, and that *T* is uniform over all FWs. This crude approximation can be improved by increasing the number of viewing chords in the future experiments. The velocity distribution of atoms in the far SOL, where the effect of the FWs is relatively larger, can then be changed. *T* and the recycling flux ratio between the inboard and outboard FWs $|\Gamma_{{\text{in}}} /\Gamma_{{\text{out}}} |$ were set as the fitting parameters. The *χ*^2^ distribution is shown in Fig. 3b. The two parameters have different effects on the spectrum shape; *T* changes the Doppler and Zeeman effects, where the latter is via the penetration depth, whereas $|\Gamma_{{\text{in}}} /\Gamma_{{\text{out}}} |$ changes the relative emissivity between the inboard and outboard SOLs. The two parameters can be determined uniquely by using both *I*_0_ and *I*_90_, and the optimized parameters were obtained as *T* = 0.15 eV and $|\Gamma_{{\text{in}}} /\Gamma_{{\text{out}}} |$= 0.9. As shown in Fig. 3b, $|\Gamma_{{\text{in}}} /\Gamma_{{\text{out}}} |$ is mainly determined by *I*_90_, while *T* is determined by both *I*_0_ and *I*_90_. This is because *I*_90_ mainly consists of the σ components and the inboard and outboard spectra can be distinguished using the difference in their Zeeman effects. By using the Stokes spectropolarimetry, the superposed π and outboard σ components can be revolved. The calculated spectra are shown with the lines in Fig. 3a. The obtained $n_{{2^{3} P}}$, which is proportional to HeI 2^3^S-2^3^P emissivity, is plotted in Fig. 2b. The 2^3^P atoms mainly exist outside the LCFS owing to an increase in ionization rate in the core region. Their velocity distribution in the plasma is determined by three factors: the *T*_e_ and *n*_e_ profiles of the background plasma, the initial velocity distribution as a function of *T* at the time of desorption, and the shape of the FWs. The last factor is due to that the emission at a certain position is produced by atoms desorbed from various locations of the FWs. Figure 4 shows the spatial variation in *f*(*v*_vc_). In the far SOL (regions a), the ionization mean free path of helium atoms is larger than the spatial scale of the plasma, and 2^3^P atoms originating from various parts of the FWs exist. Consequently, *f*(*v*_vc_) peaks near $v_{{\text{vc}}} \simeq 0$. As the atoms approach the LCFS, owing to the increase in ionization rate, atoms only having relatively large radial inward velocities survive from ionization. Eventually, in the vicinity of the LCFS, *f*(*v*_vc_) approaches the initial distribution at the FWs. The fraction of atoms ionized in the SOL was estimated to be larger in the outboard SOL owing to the larger width of the SOL, as shown in Fig. 4a. As far as the momentum exchange collisions of atoms are not significant, the atomic flux reaching the core is mainly determined by the FW shape, its distance from the plasma, and the initial velocity distribution determined by the recycling condition. Atoms not having a sufficiently large radial inward velocity are ionized in the SOL and contribute to the emissivity from the SOL but not to the core fueling.

Finally, we compared the present result with the inversion result using the two-point emission model adopted in early studies. The model regards the chord-integrated spectrum as a superposition of two spectra originating from two point light sources at the inboard and outboard SOLs. In the analysis, the chord-integrated spectrum is approximated with the expression

2 $$I(\lambda ) = \epsilon (\lambda ;s_{{({\text{in}})}} ,C_{{({\text{in}})}} ,\delta \lambda_{{{\text{D}}({\text{in}})}} ,w_{{{\text{D}}({\text{in}})}} ) + \epsilon (\lambda ;s_{{({\text{out}})}} ,C_{{({\text{out}})}} ,\delta \lambda_{{{\text{D}}({\text{out}})}} ,w_{{{\text{D}}({\text{out}})}} )$$

where *ε*(*λ*) is a function representing a spectrum shape of a point light source and is defined as

3 $$\epsilon (\lambda ;s,C,\delta \lambda_{{\text{D}}} ,w_{{\text{D}}} ) = C\sum\limits_{i} {\epsilon_{i} (s)\exp \left[ { - 4\ln 2\left( {\frac{{\lambda - \delta \lambda_{D} - \delta \lambda_{Zi} (s)}}{{w_{{\text{D}}} }}} \right)^{2} } \right]}$$

where *s* is the emission location on the viewing chord, *C* is a constant, $\delta \lambda_{\text{D}}$ and $w_{\text{D}}$ are respectively the wavelength shift and line width (FWHM) due to the Doppler effect, $\epsilon_{i}$ is the relative emissivity of the transition between the magnetic sublevels, $\delta \lambda_{\text{Z}_i}$ is the wavelength shift due to the Zeeman effect, and the summation is taken over all allowed transitions among the upper and lower magnetic sublevels. $\epsilon_{i}$ and $\delta \lambda_{\text{Z}_i}$ are functions of *s*, and the parameters are regarded as local values at the emission location. We performed a least-squares fitting of Eq. (2) simultaneously to *I*_0_ and *I*_90_. The obtained emission locations are shown in Fig. 2b,c with the red circles and red vertical lines, respectively. The parameters determined by the fitting are summarized in Table 1.

**Table 1:** Parameters obtained by fitting assuming two-point emission model.

	Inboard SOL	Outboard SOL
Intensity ratio *I*_in_/*I*_out_	0.86 ± 0.05	
Magnetic field strength (T)	2.03 ± 0.03	1.00 ± 0.02
Viewing chord velocity (km/s)	1.6 ± 0.4	− 1.7 ± 0.2
Temperature (eV)	1.18 ± 0.16	0.06 ± 0.07

The emission locations are close to the peak locations of $n_{{2^{3} P}}$. On the inboard side, the emission location is shifted toward the core, because within the constraint condition of the two-point emission model, *χ*^2^ minimum is obtained when the peak wavelength of the σ component at 1082.9 nm is slightly red-shifted, namely, with a smaller Zeeman effect. This result, however, might be spurious owing to the approximation in the emission model and noise in the spectrum, since the simultaneously determined inboard temperature of ~ 1.2 eV is significantly higher than those evaluated from the visible HeI emission lines in other devices (< ~ 0.4 eV)^22,25,27,29^. The mean viewing chord velocities of 1.6 and − 1.7 km/s and the temperature at the outboard SOL of 0.06 eV are respectively comparable to the most probable velocities of ~  ± 2 km/s and the average kinetic energy of ~ 0.1 eV of *f*(*v*_vc_) obtained by simulation in the regions b. It is thus confirmed that, as intuitively assumed, the inversion result with the two-point emission model reflects the parameters of atoms existing near the emissivity peaks.

## Conclusions

The spatial resolution of Stokes spectropolarimetry for the fusion-related toroidal plasma was improved from early studies using the two-point emission model by observing an NIR atomic emission line spectrum and using the simulation-based emission model for the inversion of the chord-integrated spectrum. The measurement was applied to the HeI 2^3^S-2^3^P emission line from a helium-puffed deuterium plasma produced in Heliotron J. Owing to the enhancement of the Zeeman effect relative to the Doppler effect, the Zeeman effect was conspicuously observed at a magnetic field strength smaller than those used in previous studies. By utilizing the Zeeman effect, we were able to reproduce the chord-integrated spectrum by simulation and the simulation result was regarded as the inversion result along the viewing chord. The apparent spatial resolution of the inversion adopted in this study is the simulation mesh size (1.25 mm × 1.25 mm), but the accuracy of the inversion result can be affected by the assumptions on the recycling flux and the accuracy of the simulation. The former can in principle be improved by increasing the number of viewing chords in future experiments, and the accuracy of the inversion may be accessed by simultaneously measuring other NIR HeI emission lines with different emissivity profiles along the viewing chord.

## Supplementary Information

Supplementary Information.

## Data Availability

The datasets used and analyzed in this study are available from the corresponding author on reasonable request.
