# Supplementary material for: Spatially resolved measurement of helium atom emission line spectrum in scrape-off layer of Heliotron J by near-infrared Stokes spectropolarimetry
Source: Sci Rep. 2022 Sep 23;12:15567. doi: 10.1038/s41598-022-19747-8 (PMC9508257; doi:10.1038/s41598-022-19747-8)
Supplement: Supplementary file 1 — Supplementary Information. [file 41598_2022_19747_MOESM1_ESM.pdf]

Supplemental information of "Spatially resolved measurement of helium atom emission line spectrum in scrape-off layer of Heliotron J by near-infrared Stokes spectropolarimetry"

Tomoki Chatani[1], Taiichi Shikama[1]*, Yohei Ueno[1], Shinichiro Kado[2], Hayato Kawazome[3], Takashi Minami[2], Ryota Matoike[4], Minato Murakumo[1], Shinji Kobayashi[2], Shinsuke Ohshima[2], Akihiro Iwata[4], Tetsutaro Oishi[5], Akihiro Ishizawa[4], Yuji Nakamura[4], Hiroyuki Okada[2], Shigeru Konoshima[2], Tohru Mizuuchi[2], Kazunobu Nagasaki[2], and Masahiro Hasuo[1]

1. Zeeman effect on HeI 2^3S-2^3P emission line spectrum

Figure S1 shows the π and σ components of the HeI 2^3S-2^3P emission line spectrum from a helium glow discharge tube (300 Pa neutral pressure) inserted in the bore of a cryogen-free superconducting magnet (Cryogenic 1721)[1]. The spectrum was measured using the Stokes spectropolarimetry system. The viewing chord was directed perpendicular to the magnetic field and the I_0 direction was aligned parallel to the magnetic field. The inhomogeneity of the field strength over the observed volume was less than 0.25%. The emission line consists of three fine structures, J-J' = 1-0, 1-1, and 1-2, with a statistical intensity ratio of 1:3:5. In the measured spectrum, the two peaks produced by 1-0 and superposed 1-1 and 1-2 transitions appear even without applying a magnetic field. When applying a magnetic field, the Zeeman effect induces the wavelength splitting of 19 transitions among the magnetic sublevels. The asymmetry in the shapes of the line peaks and σ spectra are due to the fine structure. The atomic temperature evaluated from the Doppler width is approximately 0.1 eV. Owing to the relatively high neutral pressure of the discharge tube, part of the spectrum is reabsorbed in the plasma by 2^3S metastable atoms with densities on the order of 10^{18} m^{-3}. Absorption changes the

[1]Department of Mechanical Engineering and Science, Graduate School of Engineering, Kyoto University, Kyoto 615-8540, Japan, [2]Institute of Advanced Energy, Kyoto University, Kyoto 610-0011, Japan, [3]National Institute of Technology, Kagawa Colledge, Kagawa 769-1192, Japan, [4]Graduate School of Energy Science, Kyoto University, Kyoto 610-0011, Japan, [5]National Institute for Fusion Science, Gifu 509-5292, Japan

*Corresponding author, e-mail: shikama@me.kyoto-u.ac.jp

apparent relative intensities of the 19 transitions[1,2], and the apparent intensities of the π components are smaller than those of the σ components in Fig. S1.

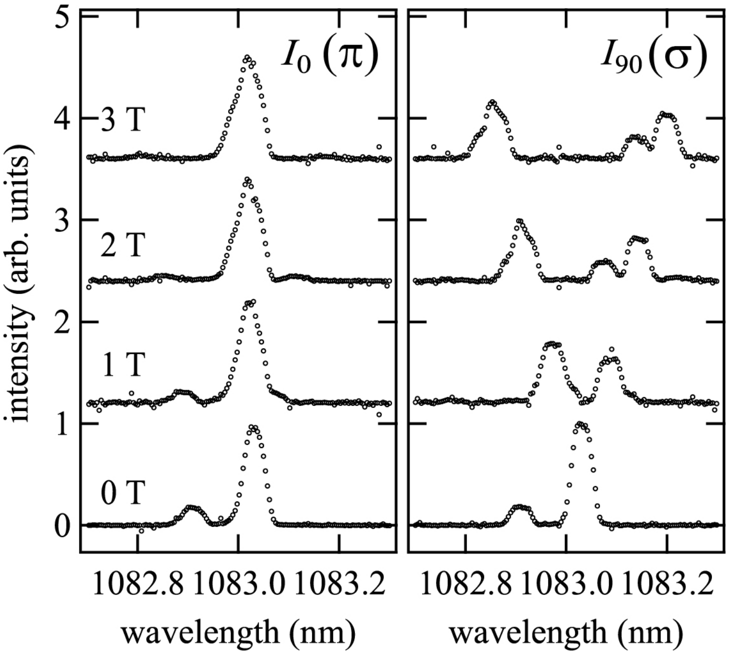

Figure S1. π and σ components of the HeI 2^3S-2^3P emission line spectrum measured in the direction perpendicular to the magnetic field in a glow discharge plasma at 300 Pa under uniform magnetic fields. The atomic temperature is evaluated to be approximately 0.1 eV. Zero levels are shifted for the spectra at 1–3 T.

2. NIR Stokes spectropolarimetry system

We used an NIR Stokes spectropolarimetry system illustrated in Fig. S2a designed for use with a single viewing chord. Emission from the plasma is decomposed to the orthogonal linear polarization components I_0 and I_{90} using a polarization beam splitter (3×10^{-3} extinction ratio). I_0 and I_{90} are transferred via different optical fibers and injected into a Czerny-Turner type spectrometer (Horiba Jovin-Yvon THR1000; 1 m focal length, 2nd-order diffraction with 720 grooves/mm grating, f/7.5). The spectrometer is equipped with an entrance double slit (100 μm width and 2.54 mm pitch), and two sets of seven bundled optical fibers (230/250 μm core/cladding diameters, 0.22 NA) separately carrying I_0 and I_{90} are aligned along the double slits. At the exit port of the spectrometer, the spectral images of I_0 and I_{90} that slightly shifted in the wavelength direction are superposed. The images are resized by 1/7 and 2.2 times in the slit and wavelength directions, respectively, using two planoconvex cylindrical lenses. The resized images are recorded with an InGaAs linear detector

(Hamamatsu G9206-512W; 0.9–2.1 μm sensitivity range, 512 pixels, 25 μm × 250 μm pixel size, -20 °C, 15 bit).

For the InGaAs linear detector, we evaluated the standard deviation of the dark noise for all the pixels and excluded flawed pixels having standard deviations that are significantly larger than statistically expected values. Figure S2b shows a histogram of the standard deviation evaluated using nine consecutive frames measured with the exposure time of 120 ms without light injection. Given that the dark noise follows a normal distribution, its standard deviation follows a Gamma distribution[3]. We fitted the histogram using a Gamma distribution with $N = 9$, where N is the number of samples. The fitting curve is shown in the figure. 72 pixels having a large standard deviation in the range of the significant level of 0.02 of the fitting curve were excluded from analysis.

The intensities of the measured I_0 and I_{90} were absolutely calibrated using diffuse reflected light from a stabilized Globar lamp (Thorlabs SLS203LM). We pre-calibrated the spectral irradiance of the lamp and regarded the diffuse reflected light unpolarized. The calibration accuracy was confirmed using the ratio I_{90}/I_0 of the HeI 2^3S-2^3P spectrum measured at 3 T shown in Fig. S1. In the wavelength range 1083.00–1083.05 nm, the spectrum has only the π component and I_{90}/I_0 can be regarded as the extinction ratio. As shown in Fig. S2c, the average ratio is $9(2) \times 10^{-3}$, where the number in the bracket denotes the standard error. The evaluated extinction ratio is smaller than the dark noise in Fig. 3a, which is $\sim 2\times 10^{-2}$ in the scale of the figure, and we neglected mixing of I_0 and I_{90} in the analysis.

The wavelengths of the measured I_0 and I_{90} were absolutely calibrated using a HeI 2^3S-2^3P emission line spectrum from a helium glow discharge lamp without the magnetic field (Electro-Technic Products). The given wavelengths at the two fine structure peaks were used neglecting their Doppler shifts. A NeI emission line spectrum of a neon glow discharge lamp (Electro-Technic Products) was regarded as the instrumental functions and the instrumental width was approximately 45 pm for both I_0 and I_{90}.

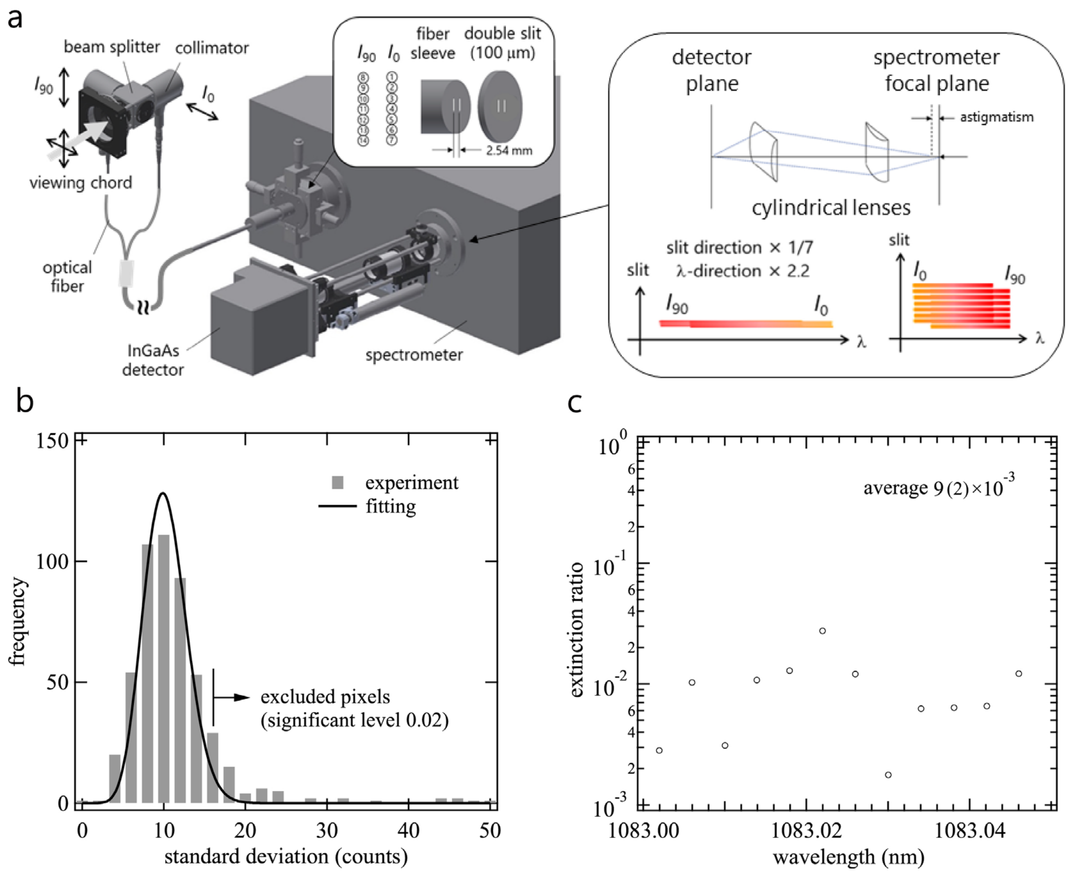

Figure S2. (**a**) Schematic illustration of an NIR Stokes spectropolarimetry system for simultaneous measurements of I_0 and I_{90} components with a single viewing chord. (**b**) Histogram of the standard deviation of the dark noise for 512 InGaAs linear detector pixels evaluated using nine consecutive frames measured with the exposure time of 120 ms without light injection. (**c**) Ratio of I_{90} (σ) to I_0 (π) components of HeI 2^3S-2^3P emission line spectrum measured at 3 T shown in Fig. S1.

3. Definition of polar and azimuthal angles

We define the polar angle γ and azimuthal angle χ of the magnetic field $\boldsymbol{B}$ with respect to the observer as illustrated in Fig. S3.

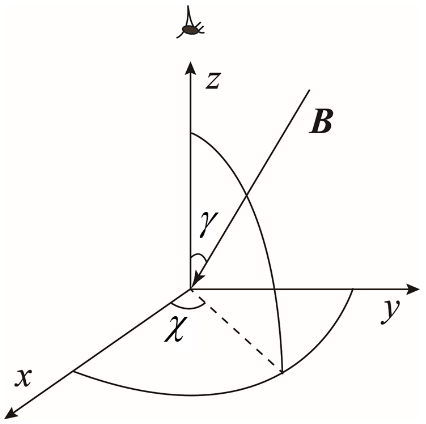

Figure S3. Definition of the polar angle γ and azimuthal angle χ with respect to the magnetic field $\boldsymbol{B}$ and the observation direction $-\hat{z}$.

4. EMC3-EIRENE calculation of the three-dimensional profiles of T_e and n_e for a pure deuterium plasma

An EMC3-EIRENE code customized for Heliotron J[4,5] was used. The input parameters were set to the values listed in Table S1. The heating power P was determined from the experimental condition. The edge electron density at r/a = 0.8, where r is the minor radius and a is that at the LCFS, and thermal diffusion coefficient χ were determined so as to minimize the differences in T_e and n_e between the calculated results and the results of measurements by Thomson scattering in the #3.5 poloidal plane as shown in Fig. S4a. The particle diffusion coefficient D was set to the previously determined value[4]; its variation does not largely affect the T_e and n_e profiles in the SOL of Heliotron J. The RZ profiles of T_e and n_e in the #10.5 poloidal plane obtained by the simulation are shown in Fig. S4b. The calculated T_e and n_e agreed with the measurements within their error bars at the inboard SOL (low-field side), while they are overestimated by a factor of ~2 at the outboard SOL (high-field side). Since the magnetic field lines at the outboard SOL in the #3.5 poloidal plane are connected to those at the inboard SOL in the #10.5 poloidal plane, the calculated T_e and n_e profiles at the inboard SOL in Fig. S4b can be overestimated. The overestimated T_e and n_e will reduce the penetration depth of atoms and to compensate for the effect, T can be overestimated.

Table S1. Adopted values of input parameters

Input parameter	Value
Heating power P (kW)	200
Edge electron density n_e at r/a = 0.8 (m^{-3})	1.0×10^{19}
Thermal diffusion coefficient χ (m^2s^{-1})	2.5
Particle diffusion coefficient D (m^2s^{-1})	0.5

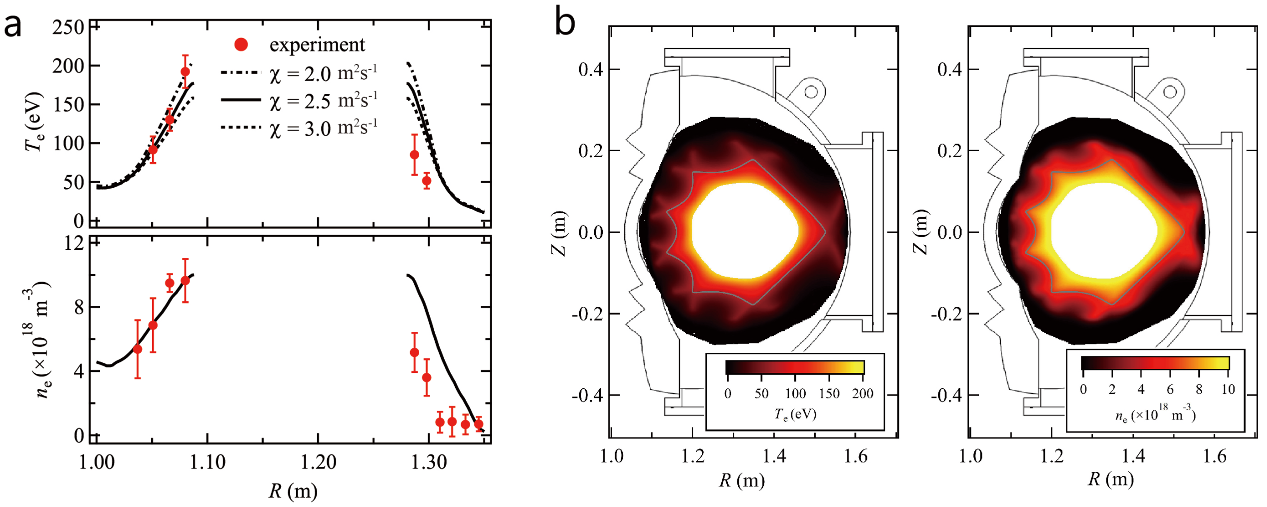

Figure S4. (**a**) Profiles of T_e and n_e in #3.5 poloidal plane calculated by EMC3-EIRENE simulation and measured by Thomson scattering. (**b**) Calculated *RZ* profiles of T_e and n_e in #10.5 poloidal plane. The black area is the calculation region.

5. Monte Carlo particle transport calculation of two-dimensional profiles of the density n_{2^3P} and velocity distribution $f(v_{vc})$ of the helium 2^{3}P excited atoms in #10.5 poloidal plane

The calculation was conducted in the #10.5 poloidal plane using a 2d3v space. The calculation region in real space is the same as that of EMC3-EIRNENE, which is in the ranges of $1.06 \le R \le 1.61$ m and $-0.4 \le Z \le 0.4$ m (the area in grey in Fig. S5a). The periodic boundary condition was assumed in the perpendicular direction. The calculation region is divided into square meshes of 1.25 mm $\times$ 1.25 mm. For helium atoms, the electronic ground (2^{1}S) and excited states included in the collisional-radiative (CR) model code[6] were considered. The calculation was carried out according to the flowchart shown in Fig. S5b. An atom is produced by desorption from the FW (Procedure 1), and its position (Procedure 2) and excited state (Procedure 3) are calculated until it is lost by ionization or reaches on other part of the FW. The velocity is assumed to be invariant from the initial velocity determined at the time of desorption.

Procedure 1: The ground-state atoms are desorbed from 72 poloidally equally spaced points on the FWs shown in Fig. S5a. The number of atoms is set to 1.0×10^5 at the point of the inboard midplane, where R = 1.06 m and Z = 0 m, and the numbers of atoms from the other points are determined so as to satisfy the predetermined recycling flux ratio $|\Gamma_{\mathrm{in}} / \Gamma_{\mathrm{out}}|$. The total number of atoms was determined to be sufficiently large in the sense that the calculation result is

reproducible. The atoms are assumed to have initial velocities following a distribution expressed as

$$f(v,\theta)dvd\Omega = \frac{1}{\pi} f_v(v)\cos\theta dvd\Omega\,, \qquad \text{(S1)}$$

where v is the velocity, θ is the polar angle of the velocity with respect to the surface normal, $d\Omega = \sin\theta d\theta d\phi$ is the solid angle of the velocity made by θ (= 0–π/2) and the azimuthal angle ϕ (= 0–2π), and $f_v(v)$ is the "v^3" T-Maxwell velocity distribution[7] defined as

$$f_v(v) = \left(\frac{m^2}{2{k_\mathrm{B}}^2 T^2}\right) v^3 \exp\left(-\frac{mv^2}{2k_\mathrm{B} T}\right), \qquad \text{(S2)}$$

where m is the mass of atoms and k_B is the Boltzmann constant. The ϕ-component of the velocity was assumed to be uniformly distributed.

Procedure 2: The total reaction rate Σ_p for an atom in the excited state p is calculated, taking into account the electron inelastic collisions and radiation using the expression

$$\Sigma_p = \sum_{q \neq p} \left(C_{pq} + F_{pq} \right) n_\mathrm{e} + S_p n_\mathrm{e} + \sum_{q<p} A_{pq}\,, \qquad \text{(S3)}$$

where q is an index of an excited state, C_{pq} and F_{pq} are the electron-impact excitation and de-excitation rate coefficients, respectively, S_p is the electron-impact ionization rate coefficient, and A_{pq} is the spontaneous emission coefficient. C_{pq}, F_{pq}, and S_p are the functions of T_e and n_e at the position of the previous step x_i. The data of C_{pq}, F_{pq}, S_p, and A_{pq} are taken from the CR-model code[6]. The mean free path of the atoms Δx_i is evaluated using the expression

$$\Delta x_i = -\ln(\xi) \frac{v}{\Sigma_p(x_i)}\,, \qquad \text{(S4)}$$

where ξ is the uniform random variable ($0 \le \xi \le 1$) and $\Sigma_p(x_i)$ is the total reaction rate at x_i. The position of the atom is then translated in the direction of its velocity by a distance Δx_i.

Procedure 3: After Procedure 2, the state of the atom is changed following the reaction rate of the inelastic collisions and radiation. The excited state p is changed to another state q $(p \neq q)$ according to the relative reaction rates at $x_{i+1} = x_i + \Delta x_i$. Then, the excited state and velocity of the atom are recorded at the nearest grid.

After the calculation of all the atoms, n_{2^3P}, $f(v_\mathrm{vc})$, and HeI 2^3S-2^3P emissivity and spectrum shape were evaluated using the total number of 2^3P excited atoms and their velocities recorded at each grid.

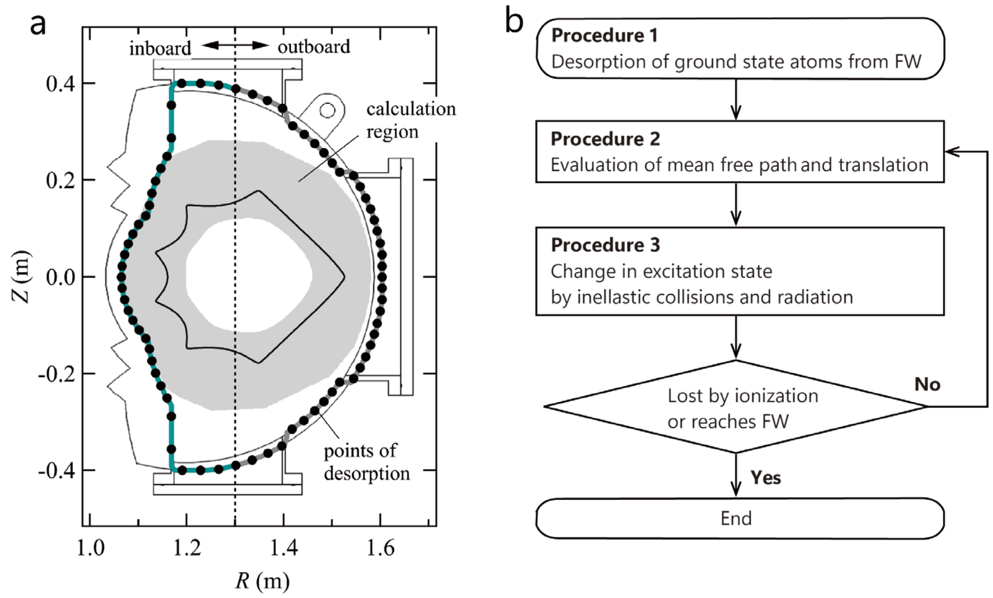

Figure S5. (**a**) Calculation region in #10.5 poloidal plane (grey area) and 72 points where atoms are desorbed from the FW (dots). (**b**) Flowchart of Monte Carlo simulation.

6. Mean free path of momentum exchange collision with electrons

The momentum exchange collision of helium atoms with electrons randomizes the atomic velocity orientation and increases the atomic temperature. An increase in the collision frequency reduces the effect of the FWs on the emissivity and velocity distribution of the atoms in the SOL. In Fig. S6, the mean free path of the collision evaluated using Eq. (S5) is plotted:

$$\bar{\lambda}_{\mathrm{m}} = \frac{\bar{v}}{n_e \sigma_{\mathrm{m}} \, | \bar{v} - \bar{v}_{\mathrm{e}} |}, \qquad \text{(S5)}$$

where $\bar{v}$ and $\bar{v}_{\mathrm{e}}$ are the mean velocities of the atoms and electrons, respectively, and σ_{m} is the cross section of the momentum exchange collision[8-10]. The evaluated mean free path is in the range $\bar{\lambda}_{\mathrm{m}} \geq\sim 0.01$ m indicating the occurrence of several times of the collisions in the SOL. The mean energy transferred from an electron to an atom per collision is given as

$$\Delta E = \frac{2m_{\mathrm{e}}}{m} E \frac{\sigma_{\mathrm{m}}}{\sigma_{\mathrm{el}}}, \qquad \text{(S6)}$$

where m_{e} and m are the masses of the electron and helium atom, respectively, E is the electron kinetic energy before the collision, and σ_{el} is the elastic-scattering cross section[11]. ΔE is approximately 9 meV at $E = 50$ eV, and the total energy transferred to the atoms in the SOL will be a fraction of the determined atomic temperature at the FWs $T = 0.15$ eV.

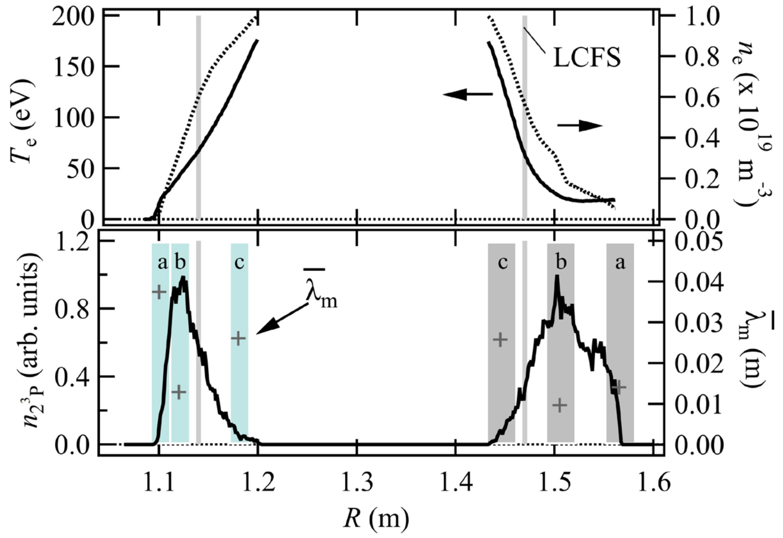

Figure S6. Same as Fig. 4a with plotting mean free path of electron momentum exchange collision $\bar{\lambda}_m$.

References

1 Shikama, T., Ogane, S., Iida, Y. & Hasuo, M. Measurement of the helium 2^3S metastable atom density by observation of the change in the 2^3S-2^3P emission line shape due to radiation reabsorption. *Journal of Physics D: Applied Physics* **49**, 025206 (2016).

2 Shikama, T., Ogane, S., Ishii, H., Iida, Y. & Hasuo, M. Measurements of helium 2^3S metastable atom density in low-pressure glow discharge plasmas by self-absorption spectroscopy of HeI 2^3S-2^3P transition. *Japanese Journal of Applied Physics* **53**, 086101 (2014).

3 Kenney, J. F. & Keeping, E. S. *The Distribution of the Standard Deviation*. 170-173 (Van Nostrand, 1951).

4 Matoike, R. *et al.* First application of 3D peripheral plasma transport code EMC3-EIRENE to Heliotron J. *Plasma and Fusion Research* **14**, 3403127 (2019). https://doi.org:10.1585/pfr.14.3403127

5 Matoike, R. *et al.* Numerical analysis of heat load distribution in Heliotron J with magnetic field tracing and plasma transport modeling. *Plasma Physics and Controlled Fusion* **63**, 115002 (2021). https://doi.org:10.1088/1361-6587/ac2069

6 Goto, M. Collisional-radiative model for neutral helium in plasma revisited. *Journal of Quantitative Spectroscopy and Radiative Transfer* **76**, 331-344 (2003).

7 Comsa, G. & David, R. Dynamical parameters of desorbing molecules. *Surf. Sci. Rep.* **5**, 145-198 (1985).

8 Carbone, E. *et al.* Data needs for modeling low-temperature non-equilibrium plasmas: the LXCat project, history, perspectives and a tutorial. *Atoms* **9**, 16 (2021).

9 Pitchford, L. C. *et al.* Lxcat: An open‐access, web‐based platform for data needed for modeling low temperature plasmas. *Plasma Processes and Polymers* **14**, 1600098 (2017).

10 Pancheshnyi, S. *et al.* The LXCat project: Electron scattering cross sections and swarm parameters for low temperature plasma modeling. *Chemical Physics* **398**, 148-153 (2012).

11 Powell, C. J., Jablonski, A., Salvat, F. & Lee, A. Y. NIST electron elastic-scattering cross-section database, version 4.0. (2016).
